# Supplementary material for: Strong confinement of active microalgae leads to inversion of vortex flow and enhanced mixing
Source: eLife. 2021 Nov 22;10:e67663. doi: 10.7554/eLife.67663 (PMC8758135; doi:10.7554/eLife.67663)
Supplement: Source code 1. [file elife-67663-supp1.zip › Source Code file.docx]

**MATLAB Source Code file**

This document contains the custom-written MATLAB codes and functions for fitting an ellipse to the edge of a *Chlamydomonas* cell (in phase-contrast imaging) and identifying the major axis vertex of the cell, located between the two flagella.

The following codes and functions are given.

1. RidgeDetection_MajorAxisVertex_AutoShift.m 🡪 When the actual major axis is at a constant offset angle to the automatically detected major axis vertex of the ellipse.
2. RidgeDetection_MajorAxisVertex_UserInput.m 🡪 When user input is needed to detect the major axis vertex.

These are the main codes to fit an ellipse to the cell edge and detect the major axis vertex (either automatically with a constant shift or using user input). The input to both these files is the binary image in which the edge of the CR cell is a black line, detected using the Ridge detection plugin in ImageJ (*Wagner and Hines, 2017*), as mentioned in the Methods.

Functions used in these codes:

1. fitellipse.m (adapted from [*https://in.mathworks.com/matlabcentral/fileexchange/15125-fitellipse-m*](https://in.mathworks.com/matlabcentral/fileexchange/15125-fitellipse-m)) 🡪 fits ellipse to the pixelated edge of CR using least squares method.
2. EllipsePlot.m 🡪 plots an ellipse when centre, major & minor axes lengths and tilt angle are given.
3. **RidgeDetection_MajorAxisVertex_AutoShift.m**

%% Obtaining ridge detected points from image and fitting ellipse

%% When the actual major axis is at a constant offset angle to the automatically detected major axis vertex of the ellipse

clear all; close all;

FS = 'fontsize'; MS = 'markersize'; LW = 'linewidth';

warning off;

tic;

%%%% Image file for ridge detection

basefile='H:\Data\2017\May17\PIV_live cells_17May17\Sample_1_200nm beads\Moving cell\MHS6_500fps_cr_ridge';

ImgFile=dir(fullfile(basefile,'*.tif'));

lastframe=length(ImgFile);

%%%% Grayscale image file

basefile_act='H:\Data\2017\May17\PIV_live cells_17May17\Sample_1_200nm beads\Moving cell\MHS6_500fps_cr';

ImgFile_act=dir(fullfile(basefile_act,'*.tif'));

lastframe_act=length(ImgFile_act);

%%% Saving the ellipse fitted parameters in a text file

textfilename_wr = 'RidgeEllipseFit_MovingMHS6.txt';

fileID_wr = fopen(textfilename_wr,'w'); % open and write, delete existing contents if any

formatSpec_wr = '%d %9.4f %9.4f %9.4f %9.4f %9.4f %9.4f\r\n';

EllipseVertex_1=[136,249]; % vertex of the ellipse in the first frame

for imageno=1:lastframe

clc; imageno

clearvars I0_act I0_act1 I0 I1

clearvars XY z a b alpha XY_ellipse

clearvars MajAxis delx dely MajAxisVertex1 MajAxisVertex2

clearvars sh_alpha sh_tilt sh_MajAxisVertex1 sh_MajAxisVertex2

clearvars EllipseWrite EllipseVertex_prev dist1 dist2

clearvars tempvar sh_tempvar v1 v2 phi

filename_act=strcat(basefile_act,'\',ImgFile_act(imageno).name);

I0_act=imread(filename_act);

filename=strcat(basefile,'\',ImgFile(imageno).name);

I0=imread(filename);

I1=im2bw(I0,0.8);

%%%% delete small regions less than 20 pixels

I1_1=bwareaopen(imcomplement(I1),20);

I1=imcomplement(I1_1);

% figure(1),

% set(gcf, 'Units', 'Normalized', 'OuterPosition', [0 0 1 1]);

% imshow(I1,[]);

%%%%% finding the black ridge

[XY(:,2),XY(:,1)]=find(I1==0);

% figure(2),

% set(gcf, 'Units', 'Normalized', 'OuterPosition', [0 0 1 1]);

% imshow(I0_act,[]);

% hold on; plot(XY(:,1),XY(:,2),'r.',MS,12);

% title(['Frame ',num2str(imageno)]);

%%%% fitting the ellipse, z-centre, a,b-axis, alpha-tilt angle

[z, a, b, phi] = fitellipse(XY);

if a>b

MajAxis=a;

MinAxis=b;

tilt=phi;

delx = MajAxis*cos(tilt);

dely = MajAxis*sin(tilt);

elseif a<b

MajAxis=b;

MinAxis=a;

tilt = phi+pi/2;

delx = MajAxis*cos(tilt);

dely = MajAxis*sin(tilt);

end

%%%% plotting the ellipse

XY_ellipse=EllipsePlot(z,MajAxis,MinAxis,tilt);

% hold on; plot(XY_ellipse(1,:),XY_ellipse(2,:),'y-',LW,1.5);

% plot(z(1),z(2),'m*',MS,18,LW,1.5);

MajAxisVertex1(1) = z(1)-delx; MajAxisVertex1(2) = z(2)-dely;

MajAxisVertex2(1) = z(1)+delx; MajAxisVertex2(2) = z(2)+dely;

%%%% shifted Actual Major Axis

sh_alpha=deg2rad(8);

sh_tilt=tilt-sh_alpha;

sh_MajAxisVertex1(1) = z(1)-(MajAxis*cos(sh_tilt));

sh_MajAxisVertex1(2) = z(2)-(MajAxis*sin(sh_tilt));

sh_MajAxisVertex2(1) = z(1)+(MajAxis*cos(sh_tilt));

sh_MajAxisVertex2(2) = z(2)+(MajAxis*sin(sh_tilt));

%%%% Choosing the correct ellipse vertex

if imageno==1

EllipseVertex_prev=EllipseVertex_1;

else

EllipseVertex_prev=MAV(imageno-1,:);

end

dist1=((MajAxisVertex1(1)-EllipseVertex_prev(1))^2+...

(MajAxisVertex1(2)-EllipseVertex_prev(2))^2).^0.5;

dist2=((MajAxisVertex2(1)-EllipseVertex_prev(1))^2+...

(MajAxisVertex2(2)-EllipseVertex_prev(2))^2).^0.5;

if dist2<dist1

tempvar=MajAxisVertex1;

MajAxisVertex1=MajAxisVertex2;

MajAxisVertex2=tempvar;

sh_tempvar=sh_MajAxisVertex1;

sh_MajAxisVertex1=sh_MajAxisVertex2;

sh_MajAxisVertex2=sh_tempvar;

end

MAV(imageno,:)=MajAxisVertex1;

% hold on,

% qui1=quiver(MajAxisVertex2(1),MajAxisVertex2(2),MajAxisVertex1(1)-MajAxisVertex2(1),...

% MajAxisVertex1(2)-MajAxisVertex2(2),'b',LW,2.5);

% qui1.AutoScale='off'; qui1.MaxHeadSize=0.4;

% qui2=quiver(sh_MajAxisVertex2(1),sh_MajAxisVertex2(2),sh_MajAxisVertex1(1)-sh_MajAxisVertex2(1),...

% sh_MajAxisVertex1(2)-sh_MajAxisVertex2(2),'g',LW,2.5);

% qui2.AutoScale='off'; qui2.MaxHeadSize=0.4;

%%%% Finding the angle between shifted major axis vector and +ve x axis

v1=[z(1)+MajAxis,z(2)]-[z(1),z(2)];

v2=[sh_MajAxisVertex1(1),sh_MajAxisVertex1(2)]-[z(1),z(2)];

phi=acos(dot(v1,v2)/(norm(v1)*norm(v2))); % rotation angle wrt major axis

if sh_MajAxisVertex1(2)<z(2)

phi=-phi;

end

%%%% Saving Ellipse data fit in a text file

EllipseWrite(1,1)=imageno; % Frame

EllipseWrite(1,2)=z(1); % Center

EllipseWrite(1,3)=z(2);

EllipseWrite(1,4)=MajAxis; % Major Axis length

EllipseWrite(1,5)=MinAxis; % Minor Axis length

EllipseWrite(1,6)=phi; % actual Major Axis tilt angle

EllipseWrite(1,7)=tilt; % ellipse tilt angle

%%%% printing the data in textfile

fprintf(fileID_wr,formatSpec_wr,EllipseWrite');

% pause(1);

end

% legend('Ridge Detection in ImageJ','Ellipse fit','Ellipse Centre',...

% 'Major Axis','Major Axis Vertex','Actual Major Axis','Actual Major Axis Vertex');

disp(['Elapsed time = ',num2str(toc/60),' min'])

fclose(fileID_wr);

1. **RidgeDetection_MajorAxisVertex_UserInput.m**

%% Obtaining ridge detected points from image and fitting ellipse

%%%%% Choosing the major axis vertex by user input

clear all; close all;

FS = 'fontsize'; MS = 'markersize'; LW = 'linewidth';

warning off;

tic;

Movie = '67_500fps_210to242';

%%%% Image file for ridge detection

basefile=['I:\Data\2019\Oct19\PTV_Cells_15Oct19\S3_D30_40XPh_12_03pm\',Movie,'_cr_ridge'];

ImgFile=dir(fullfile(basefile,'*.tif'));

lastframe=length(ImgFile);

%%%% Grayscale image file

basefile_act=['I:\Data\2019\Oct19\PTV_Cells_15Oct19\S3_D30_40XPh_12_03pm\',Movie];

ImgFile_act=dir(fullfile(basefile_act,'*.tif'));

lastframe_act=length(ImgFile_act);

%%% Getting the crop and MAV details

BB = readtable('Crop details.txt','Delimiter','tab');

C_ch = contains(BB{:,1},Movie);

C_idx=find(C_ch==1);

image_cr=str2num(cell2mat(BB{C_idx,2})); % crop range

EllipseVertex_1=str2num(cell2mat(BB{C_idx,3})); % vertex of the ellipse in the first frame

%%%%%% Saving the ellipse fitted parameters in a text file

textfilename_wr = [Movie,'_RidgeEllipse.txt'];

fileID_wr = fopen(['Ellipse Data\',textfilename_wr],'w'); % open and write, delete existing contents if any

fprintf(fileID_wr, '%5s\t %8s\t %8s\t %8s\t %8s\t %8s\t %8s\r\n',...

'Imageno','Center_X[pix]','Center_Y[pix]','MajAxis a[pix]','MinAxis b[pix]','MajAx_phi[rad]','Ell_phi[rad]');

formatSpec_wr = '%d\t %9.4f\t %9.4f\t %9.4f\t %9.4f\t %9.4f\t %9.4f\r\n';

for imageno=1:lastframe

clc; imageno

clearvars I0_act I0 I1

clearvars XY z a b alpha XY_ellipse

clearvars MajAxis delx dely MajAxisVertex1 MajAxisVertex2

clearvars MAV_X MAV_Y n_delx n_dely n_MAV1 n_MAV2

clearvars EllipseWrite EllipseVertex_prev dist1 dist2

clearvars tempvar v1 v2 phi

filename_act=strcat(basefile_act,'\',ImgFile_act(imageno).name);

I0_act1=imread(filename_act);

I0_act=imcrop(I0_act1,image_cr);

filename=strcat(basefile,'\',ImgFile(imageno).name);

I0=imread(filename);

I1=im2bw(I0,0.8);

%%%% delete small regions less than 10 pixels

I1_1=bwareaopen(imcomplement(I1),10);

I1=imcomplement(I1_1);

%%%%% finding the black ridge

[XY(:,2),XY(:,1)]=find(I1==0);

figure(2),

set(gcf, 'Units', 'Normalized', 'OuterPosition', [0 0 1 1]);

imshow(I0_act,[]);

hold on; plot(XY(:,1),XY(:,2),'r.',MS,10);

title(['Frame ',num2str(imageno)]);

%%%% fitting the ellipse, z-centre, a,b-axis, alpha-tilt angle

[z, a, b, alpha] = fitellipse(XY);

if a>b

MajAxis=a;

MinAxis=b;

tilt=alpha;

elseif a<b

MajAxis=b;

MinAxis=a;

tilt = alpha+pi/2;

end

%%%% plotting the ellipse

XY_ellipse = EllipsePlot(z,MajAxis,MinAxis,tilt);

hold on; plot(XY_ellipse(1,:),XY_ellipse(2,:),'y-',LW,1.5);

plot(z(1),z(2),'m*',MS,18,LW,1.5);

delx = MajAxis*cos(tilt);

dely = MajAxis*sin(tilt);

MajAxisVertex1(1) = z(1)+delx; MajAxisVertex1(2) = z(2)+dely;

MajAxisVertex2(1) = z(1)-delx; MajAxisVertex2(2) = z(2)-dely;

%%%% Choosing the correct ellipse vertex

if imageno==1

EllipseVertex_prev=EllipseVertex_1;

else

EllipseVertex_prev=MAV(imageno-1,:);

end

dist1=((MajAxisVertex1(1)-EllipseVertex_prev(1))^2+...

(MajAxisVertex1(2)-EllipseVertex_prev(2))^2).^0.5;

dist2=((MajAxisVertex2(1)-EllipseVertex_prev(1))^2+...

(MajAxisVertex2(2)-EllipseVertex_prev(2))^2).^0.5;

if dist2<dist1

tempvar=MajAxisVertex1;

MajAxisVertex1=MajAxisVertex2;

MajAxisVertex2=tempvar;

end

MAV(imageno,:)=MajAxisVertex1;

Traj_XY(imageno,1:2)=z(1:2);

hold on,

qui1=quiver(MajAxisVertex2(1),MajAxisVertex2(2),MajAxisVertex1(1)-MajAxisVertex2(1),...

MajAxisVertex1(2)-MajAxisVertex2(2),'b',LW,2);

qui1.AutoScale='off'; qui1.MaxHeadSize=0.35;

plot(Traj_XY(:,1),Traj_XY(:,2),'ko-',LW,1.5,MS,5,'MarkerFaceColor','c');

%%%%%% Choosing the major axis vertex from user input

[MAV_X,MAV_Y] = ginput(1);

%%%% Finding the angle between shifted major axis vector and +ve x axis

v1=[z(1)+MajAxis,z(2)]-[z(1),z(2)];

v2=[MAV_X,MAV_Y]-[z(1),z(2)];

phi=acos(dot(v1,v2)/(norm(v1)*norm(v2))); % rotation angle wrt major axis

if MAV_Y<z(2)

phi=-phi;

end

%%%% finding the major axis vertices

n_delx = MajAxis*cos(phi);

n_dely = MajAxis*sin(phi);

n_MAV1(1) = z(1)+n_delx; n_MAV1(2) = z(2)+n_dely;

n_MAV2(1) = z(1)-n_delx; n_MAV2(2) = z(2)-n_dely;

hold on,

qui1=quiver(n_MAV2(1),n_MAV2(2),n_MAV1(1)-n_MAV2(1),n_MAV1(2)-n_MAV2(2),'g',LW,2);

qui1.AutoScale='off'; qui1.MaxHeadSize=0.35;

%%%% Saving Ellipse data fit in a text file

EllipseWrite(1,1)=imageno; % Frame

EllipseWrite(1,2)=z(1); % Center

EllipseWrite(1,3)=z(2);

EllipseWrite(1,4)=MajAxis; % Major Axis length

EllipseWrite(1,5)=MinAxis; % Minor Axis length

EllipseWrite(1,6)=phi; % actual Major Axis tilt angle

EllipseWrite(1,7)=tilt; % ellipse tilt angle

%%% printing the data in textfile

fprintf(fileID_wr,formatSpec_wr,EllipseWrite');

end

disp(['Elapsed time = ',num2str(toc/60),' min'])

fclose(fileID_wr);

1. **fitellipse.m**

Ref: Richard Brown (2021). fitellipse.m ([*https://in.mathworks.com/matlabcentral/fileexchange/15125-fitellipse-m*](https://in.mathworks.com/matlabcentral/fileexchange/15125-fitellipse-m)), MATLAB Central File Exchange. Retrieved November 10, 2021.

Fit ellipses to 2D points using linear or nonlinear least squares

function [z, a, b, alpha] = fitellipse(x, varargin)

%FITELLIPSE least squares fit of ellipse to 2D data

%

% [Z, A, B, ALPHA] = FITELLIPSE(X)

% Fit an ellipse to the 2D points in the 2xN array X. The ellipse is

% returned in parametric form such that the equation of the ellipse

% parameterised by 0 <= theta < 2*pi is:

% X = Z + Q(ALPHA) * [A * cos(theta); B * sin(theta)]

% where Q(ALPHA) is the rotation matrix

% Q(ALPHA) = [cos(ALPHA), -sin(ALPHA);

% sin(ALPHA), cos(ALPHA)]

%

% Fitting is performed by nonlinear least squares, optimising the

% squared sum of orthogonal distances from the points to the fitted

% ellipse. The initial guess is calculated by a linear least squares

% routine, by default using the Bookstein constraint (see below)

%

% [...] = FITELLIPSE(X, 'linear')

% Fit an ellipse using linear least squares. The conic to be fitted

% is of the form

% x'Ax + b'x + c = 0

% and the algebraic error is minimised by least squares with the

% Bookstein constraint (lambda_1^2 + lambda_2^2 = 1, where

% lambda_i are the eigenvalues of A)

%

% [...] = FITELLIPSE(..., 'Property', 'value', ...)

% Specify property/value pairs to change problem parameters

% Property Values

% =================================

% 'constraint' {|'bookstein'|, 'trace'}

% For the linear fit, the following

% quadratic form is considered

% x'Ax + b'x + c = 0. Different

% constraints on the parameters yield

% different fits. Both 'bookstein' and

% 'trace' are Euclidean-invariant

% constraints on the eigenvalues of A,

% meaning the fit will be invariant

% under Euclidean transformations

% 'bookstein': lambda1^2 + lambda2^2 = 1

% 'trace' : lambda1 + lambda2 = 1

%

% Nonlinear Fit Property Values

% ===============================

% 'maxits' positive integer, default 200

% Maximum number of iterations for the

% Gauss Newton step

%

% 'tol' positive real, default 1e-5

% Relative step size tolerance

% Example:

% % A set of points

% x = [1 2 5 7 9 6 3 8;

% 7 6 8 7 5 7 2 4];

%

% % Fit an ellipse using the Bookstein constraint

% [zb, ab, bb, alphab] = fitellipse(x, 'linear');

%

% % Find the least squares geometric estimate

% [zg, ag, bg, alphag] = fitellipse(x);

%

% % Plot the results

% plot(x(1,:), x(2,:), 'ro')

% hold on

% % plotellipse(zb, ab, bb, alphab, 'b--')

% % plotellipse(zg, ag, bg, alphag, 'k')

%

% See also PLOTELLIPSE

% Copyright Richard Brown, this code can be freely used and modified so

% long as this line is retained

error(nargchk(1, 5, nargin, 'struct'))

% Default parameters

params.fNonlinear = true;

params.constraint = 'bookstein';

params.maxits = 200;

params.tol = 1e-5;

% Parse inputs

[x, params] = parseinputs(x, params, varargin{:});

% Constraints are Euclidean-invariant, so improve conditioning by removing

% centroid

centroid = mean(x, 2);

x = x - repmat(centroid, 1, size(x, 2));

% Obtain a linear estimate

switch params.constraint

% Bookstein constraint : lambda_1^2 + lambda_2^2 = 1

case 'bookstein'

[z, a, b, alpha] = fitbookstein(x);

% 'trace' constraint, lambda1 + lambda2 = trace(A) = 1

case 'trace'

[z, a, b, alpha] = fitggk(x);

end % switch

% Minimise geometric error using nonlinear least squares if required

if params.fNonlinear

% Initial conditions

z0 = z;

a0 = a;

b0 = b;

alpha0 = alpha;

% Apply the fit

[z, a, b, alpha, fConverged] = ...

fitnonlinear(x, z0, a0, b0, alpha0, params);

% Return linear estimate if GN doesn't converge

if ~fConverged

warning('fitellipse:FailureToConverge', ...'

'Gauss-Newton did not converge, returning linear estimate');

z = z0;

a = a0;

b = b0;

alpha = alpha0;

end

end

% Add the centroid back on

z = z + centroid;

end % fitellipse

% ----END MAIN FUNCTION-----------%

function [z, a, b, alpha] = fitbookstein(x)

%FITBOOKSTEIN Linear ellipse fit using bookstein constraint

% lambda_1^2 + lambda_2^2 = 1, where lambda_i are the eigenvalues of A

% Convenience variables

m = size(x, 2);

x1 = x(1, :)';

x2 = x(2, :)';

% Define the coefficient matrix B, such that we solve the system

% B *[v; w] = 0, with the constraint norm(w) == 1

B = [x1, x2, ones(m, 1), x1.^2, sqrt(2) * x1 .* x2, x2.^2];

% To enforce the constraint, we need to take the QR decomposition

[Q, R] = qr(B);

% Decompose R into blocks

R11 = R(1:3, 1:3);

R12 = R(1:3, 4:6);

R22 = R(4:6, 4:6);

% Solve R22 * w = 0 subject to norm(w) == 1

[U, S, V] = svd(R22);

w = V(:, 3);

% Solve for the remaining variables

v = -R11 \ R12 * w;

% Fill in the quadratic form

A = zeros(2);

A(1) = w(1);

A([2 3]) = 1 / sqrt(2) * w(2);

A(4) = w(3);

bv = v(1:2);

c = v(3);

% Find the parameters

[z, a, b, alpha] = conic2parametric(A, bv, c);

end % fitellipse

function [z, a, b, alpha] = fitggk(x)

% Linear least squares with the Euclidean-invariant constraint Trace(A) = 1

% Convenience variables

m = size(x, 2);

x1 = x(1, :)';

x2 = x(2, :)';

% Coefficient matrix

B = [2 * x1 .* x2, x2.^2 - x1.^2, x1, x2, ones(m, 1)];

v = B \ -x1.^2;

% For clarity, fill in the quadratic form variables

A = zeros(2);

A(1,1) = 1 - v(2);

A([2 3]) = v(1);

A(2,2) = v(2);

bv = v(3:4);

c = v(5);

% find parameters

[z, a, b, alpha] = conic2parametric(A, bv, c);

end

function [z, a, b, alpha, fConverged] = fitnonlinear(x, z0, a0, b0, alpha0, params)

% Gauss-Newton least squares ellipse fit minimising geometric distance

% Get initial rotation matrix

Q0 = [cos(alpha0), -sin(alpha0); sin(alpha0) cos(alpha0)];

m = size(x, 2);

% Get initial phase estimates

phi0 = angle( [1 i] * Q0' * (x - repmat(z0, 1, m)) )';

u = [phi0; alpha0; a0; b0; z0];

% Iterate using Gauss Newton

fConverged = false;

for nIts = 1:params.maxits

% Find the function and Jacobian

[f, J] = sys(u);

% Solve for the step and update u

h = -J \ f;

u = u + h;

% Check for convergence

delta = norm(h, inf) / norm(u, inf);

if delta < params.tol

fConverged = true;

break

end

end

alpha = u(end-4);

a = u(end-3);

b = u(end-2);

z = u(end-1:end);

function [f, J] = sys(u)

% SYS : Define the system of nonlinear equations and Jacobian. Nested

% function accesses X (but changeth it not)

% from the FITELLIPSE workspace

% Tolerance for whether it is a circle

circTol = 1e-5;

% Unpack parameters from u

phi = u(1:end-5);

alpha = u(end-4);

a = u(end-3);

b = u(end-2);

z = u(end-1:end);

% If it is a circle, the Jacobian will be singular, and the

% Gauss-Newton step won't work.

%TODO: This can be fixed by switching to a Levenberg-Marquardt

%solver

if abs(a - b) / (a + b) < circTol

warning('fitellipse:CircleFound', ...

'Ellipse is near-circular - nonlinear fit may not succeed')

end

% Convenience trig variables

c = cos(phi);

s = sin(phi);

ca = cos(alpha);

sa = sin(alpha);

% Rotation matrices

Q = [ca, -sa; sa, ca];

Qdot = [-sa, -ca; ca, -sa];

% Preallocate function and Jacobian variables

f = zeros(2 * m, 1);

J = zeros(2 * m, m + 5);

for i = 1:m

rows = (2*i-1):(2*i);

% Equation system - vector difference between point on ellipse

% and data point

f((2*i-1):(2*i)) = x(:, i) - z - Q * [a * cos(phi(i)); b * sin(phi(i))];

% Jacobian

J(rows, i) = -Q * [-a * s(i); b * c(i)];

J(rows, (end-4:end)) = ...

[-Qdot*[a*c(i); b*s(i)], -Q*[c(i); 0], -Q*[0; s(i)], [-1 0; 0 -1]];

end

end

end % fitnonlinear

function [z, a, b, alpha] = conic2parametric(A, bv, c)

% Diagonalise A - find Q, D such at A = Q' * D * Q

[Q, D] = eig(A);

Q = Q';

% If the determinant < 0, it's not an ellipse

if prod(diag(D)) <= 0

error('fitellipse:NotEllipse', 'Linear fit did not produce an ellipse');

end

% We have b_h' = 2 * t' * A + b'

t = -0.5 * (A \ bv);

c_h = t' * A * t + bv' * t + c;

z = t;

a = sqrt(-c_h / D(1,1));

b = sqrt(-c_h / D(2,2));

alpha = atan2(Q(1,2), Q(1,1));

end % conic2parametric

function [x, params] = parseinputs(x, params, varargin)

% PARSEINPUTS put x in the correct form, and parse user parameters

% CHECK x

% Make sure x is 2xN where N > 3

if size(x, 2) == 2

x = x';

end

if size(x, 1) ~= 2

error('fitellipse:InvalidDimension', ...

'Input matrix must be two dimensional')

end

if size(x, 2) < 6

error('fitellipse:InsufficientPoints', ...

'At least 6 points required to compute fit')

end

% Determine whether we are solving for geometric (nonlinear) or algebraic

% (linear) distance

if ~isempty(varargin) && strncmpi(varargin{1}, 'linear', length(varargin{1}))

params.fNonlinear = false;

varargin(1) = [];

else

params.fNonlinear = true;

end

% Parse property/value pairs

if rem(length(varargin), 2) ~= 0

error('fitellipse:InvalidInputArguments', ...

'Additional arguments must take the form of Property/Value pairs')

end

% Cell array of valid property names

properties = {'constraint', 'maxits', 'tol'};

while length(varargin) ~= 0

% Pop pair off varargin

property = varargin{1};

value = varargin{2};

varargin(1:2) = [];

% If the property has been supplied in a shortened form, lengthen it

iProperty = find(strncmpi(property, properties, length(property)));

if isempty(iProperty)

error('fitellipse:UnknownProperty', 'Unknown Property');

elseif length(iProperty) > 1

error('fitellipse:AmbiguousProperty', ...

'Supplied shortened property name is ambiguous');

end

% Expand property to its full name

property = properties{iProperty};

% Check for irrelevant property

if ~params.fNonlinear && ismember(property, {'maxits', 'tol'})

warning('fitellipse:IrrelevantProperty', ...

'Supplied property has no effect on linear estimate, ignoring');

continue

end

% Check supplied property value

switch property

case 'maxits'

if ~isnumeric(value) || value <= 0

error('fitcircle:InvalidMaxits', ...

'maxits must be an integer greater than 0')

end

params.maxits = value;

case 'tol'

if ~isnumeric(value) || value <= 0

error('fitcircle:InvalidTol', ...

'tol must be a positive real number')

end

params.tol = value;

case 'constraint'

switch lower(value)

case 'bookstein'

params.constraint = 'bookstein';

case 'trace'

params.constraint = 'trace';

otherwise

error('fitellipse:InvalidConstraint', ...

'Invalid constraint specified')

end

end % switch property

end % while

end % parseinputs

1. **EllipsePlot.m**

Input to this file: ‘C’ as centre; ‘a’ and ‘b’ as major and minor axes lengths, ‘phi’ as the tilt angle of the ellipse

function XY_ellipse = EllipsePlot(C,a,b,phi)

theta=linspace(0,2*pi,100);

XY_ellipse(1,:)=C(1)+ (a*cos(theta).*cos(phi) - b*sin(theta).*sin(phi));

XY_ellipse(2,:)=C(2)+ (a*cos(theta).*sin(phi) + b*sin(theta).*cos(phi));

end


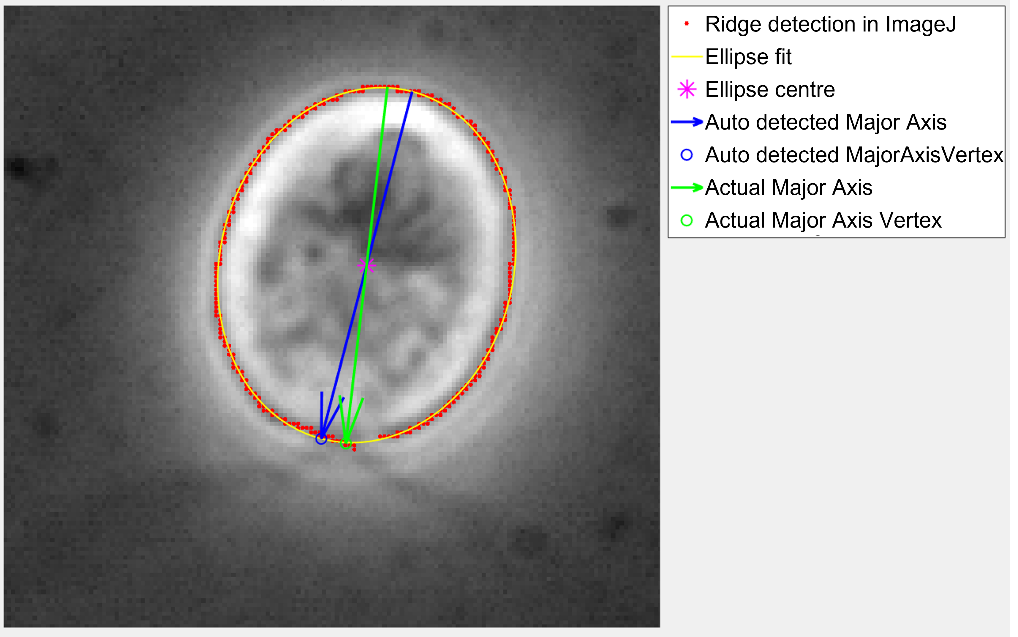


Figure: A representative image of a *Chlamydomonas* cell in phase-contrast imaging. Legends indicate corresponding symbols. The auto-detected major axis of the ellipse (blue arrow) and its vertex (blue circle) do not indicate the actual orientation of the cell. The orientation is actually denoted by the line joining the centre of the cell to the point at the edge of the cell where the two flagella originate (green circle). Therefore, green arrow refers to the actual major axis of the cell.
