# Supplementary material for: Strong confinement of active microalgae leads to inversion of vortex flow and enhanced mixing
Source: eLife. 2021 Nov 22;10:e67663. doi: 10.7554/eLife.67663 (PMC8758135; doi:10.7554/eLife.67663)
Supplement: Transparent reporting form [file elife-67663-transrepform1.docx]

***eLife’s* transparent reporting form**

We encourage authors to provide detailed information *within their submission* to facilitate the interpretation and replication of experiments. Authors can upload supporting documentation to indicate the use of appropriate reporting guidelines for health-related research (see [EQUATOR Network](http://www.equator-network.org/%20)), life science research (see the [BioSharing Information Resource](https://biosharing.org/)), or the [ARRIVE guidelines](http://www.plosbiology.org/article/info:doi/10.1371/journal.pbio.1000412) for reporting work involving animal research. Where applicable, authors should refer to any relevant reporting standards documents in this form.

If you have any questions, please consult our Journal Policies and/or contact us: [editorial@elifesciences.org](mailto:editorial@elifesciences.org).

**Sample-size estimation**

- You should state whether an appropriate sample size was computed when the study was being designed
- You should state the statistical method of sample size computation and any required assumptions
- If no explicit power analysis was used, you should describe how you decided what sample (replicate) size (number) to use

Please outline where this information can be found within the submission (e.g., sections or figure legends), or explain why this information doesn’t apply to your submission:

This information is mentioned in figure legends or in the text associated with the figures in the ‘Results’ section. In addition, ‘Materials and Methods’ section also contains this information wherever appropriate.

**Replicates**

- You should report how often each experiment was performed
- You should include a definition of biological versus technical replication
- The data obtained should be provided and sufficient information should be provided to indicate the number of independent biological and/or technical replicates
- If you encountered any outliers, you should describe how these were handled
- Criteria for exclusion/inclusion of data should be clearly stated
- High-throughput sequence data should be uploaded before submission, with a private link for reviewers provided (these are available from both GEO and ArrayExpress)

Please outline where this information can be found within the submission (e.g., sections or figure legends), or explain why this information doesn’t apply to your submission:

All relevant information is mentioned in the second paragraph of subsection ‘Sample imaging’ in the ‘Materials and Methods’ section.

Criteria for exclusion/inclusion of data for Figure 2G are mentioned in the ‘Trajectory tortuosity’ subsection in ‘Materials and Methods’.

**Statistical reporting**

- Statistical analysis methods should be described and justified
- Raw data should be presented in figures whenever informative to do so (typically when N per group is less than 10)
- For each experiment, you should identify the statistical tests used, exact values of N, definitions of center, methods of multiple test correction, and dispersion and precision measures (e.g., mean, median, SD, SEM, confidence intervals; and, for the major substantive results, a measure of effect size (e.g., Pearson's r, Cohen's d)
- Report exact p-values wherever possible alongside the summary statistics and 95% confidence intervals. These should be reported for all key questions and not only when the p-value is less than 0.05.

Please outline where this information can be found within the submission (e.g., sections or figure legends), or explain why this information doesn’t apply to your submission:

This information is mentioned in the ‘Results’ section of the manuscript (mean ± standard deviation due to heterogeneous population of cells).

(For large datasets, or papers with a very large number of statistical tests, you may upload a single table file with tests, Ns, etc., with reference to sections in the manuscript.)

**Group allocation**

- Indicate how samples were allocated into experimental groups (in the case of clinical studies, please specify allocation to treatment method); if randomization was used, please also state if restricted randomization was applied
- Indicate if masking was used during group allocation, data collection and/or data analysis

Please outline where this information can be found within the submission (e.g., sections or figure legends), or explain why this information doesn’t apply to your submission:

This is not applicable for our study.

**Additional data files (“source data”)**

- We encourage you to upload relevant additional data files, such as numerical data that are represented as a graph in a figure, or as a summary table
- Where provided, these should be in the most useful format, and they can be uploaded as “Source data” files linked to a main figure or table
- Include model definition files including the full list of parameters used
- Include code used for data analysis (e.g., R, MatLab)
- Avoid stating that data files are “available upon request”

Please indicate the figures or tables for which source data files have been provided:

We have uploaded the source data file for all Figures (except the raw image files and Figure 5, B and C) in separate excel files and each excel file is to linked to a subfigure as denoted in the Figure captions of the main manuscript. We have cited the standard plugins and MATLAB tracking functions that we use in the ‘Particle Tracking Velocimetry’ subsection in Methods. We have also provided the custom-written MATLAB codes and functions for fitting an ellipse to the edge of a Chlamydomonas cell and identifying its major axis vertex in a separate source code file ‘Source Code file.docx’. All data analysis methods are presented in the main text and in Materials and Methods. The numerical computation of Inverse Fourier Transform is detailed in Appendix 1, section 3.

The source movies are of large size (each movie about 10 GB) due to high-speed recording and are available upon request. Similarly, Figure 5, B and C contain trajectories of over 600 tracers, each and hence, the data file is very large for each one of them. They are available upon request.
